# LncRNA ZFAS1 regulates the proliferation, oxidative stress, fibrosis, and inflammation of high glucose-induced human mesangial cells via the miR-588/ROCK1 axis

**DOI:** 10.1186/s13098-022-00791-3

**Published:** 2022-01-28

**Authors:** Zhuang Geng, Bingzi Dong, Wenshan Lv, Zhongchao Wang, Xiang Wang, YaJing Huang, Yangang Wang, Lili Xu

**Affiliations:** Department of Endocrinology and Metabolism, The Affiliated Hospital of Qingdao University, Qingdao, 266000 Shandong People’s Republic of China

**Keywords:** Diabetic nephropathy, Mesangial cells, Fibrosis, ZFAS1, miR-588, ROCK1

## Abstract

**Background:**

Diabetic nephropathy (DN) is a critical and the most common microvascular complication and its pathogenesis is still faintly understood. Thus, this study was performed to examine the long non-coding RNA ZNFX1 Antisense Gene Protein 1 (lncRNA ZFAS1) biological function and mechanism of regulation in DN.

**Method:**

Human glomerular mesangial cells (HGMC) were induced with high glucose (HG, 25 mM) to establish HG-induced cell viability, pro-inflammation observed in DN. After, target miRNA and mRNA were predicted through Lncbase and Targetscan. Subsequently, the expression of ZFAS1, miR-588, and ROCK1 in DN clinical samples and cell-model was examined through qRT-PCR and western blot analysis. We upheld the targeted interaction between miR-588 and ZFAS1 or ROCK1 through a dual-luciferase reporter assay. The proliferation of the cell was also examined through CCK-8 assay, while the level of HG-induced oxidative stress was established by measuring reactive oxygen species (ROS) level, and also the activities of antioxidant enzymes in the cell. Lastly, the level of accumulated extracellular matrix (ECM) protein-fibronectin and collagen type IV, and inflammatory cytokines produced by the cell was analyzed through western blot analysis and ELISA.

**Results:**

ZFAS1 was significantly upregulated in the DN blood samples and HG-induced HGMC. Prediction result revealed that the ZFAS1 endogenously targets the miR-588 seed sequence while miR-588 plays a role in post-transcriptional regulation of ROCK1 mRNA. Moreover, we found that miR-588 expression was significantly downregulated in DN blood samples and negatively correlates with ZFAS1 expression. Further results show that silencing ZFAS1 had a protective effect on HG-induced proliferation, oxidative stress, fibrosis, and inflammation in HGMC while miR-588 inhibition and ROCK1 overexpression reversed this effect.

**Conclusions:**

Altogether, our data suggest that ZFAS1 regulates the proliferation, oxidative stress, fibrosis, and inflammation of high glucose-induced diabetic nephropathy through the miR-588/ROCK1 axis.

## Introduction

Diabetic nephropathy (DN), a critical and the most common microvascular complication of diabetes mellitus, remains the leading cause of end-stage renal disease in the US and also in China [1, 2]. Although its pathogenesis is complex and has not been fully understood, it is majorly associated with type 2 diabetes (T2DM) and is characterized by a pathological increase in the level of urinary albumin, renal fibrosis, inflammation, and glomerular lesions which causes a reduction in glomerular filtration rate (GFR) in patients [3]. Furthermore, accumulating evidence shows that the pooled prevalence of DN in China is about 21.8%, and its treatment remains ineffective while its diagnosis is always late [1]. Moreover, several studies have shown that the mutation or knockdown of numerous genes could be responsible for the progression of the disease. For instance, Xu et al. demonstrated that a deletion of the SMAD3 gene could transcriptionally inhibit lncRNA Erbb4-IR, consequently, increase miR-29b transcription, and protect the kidney from progressive renal injury in DN [4]. Contrarily, a conditional knockout of HIF-1α was reported to worsen the tubular injury, exacerbate kidney dysfunction, and lead to the accumulation of reactive oxygen species (ROS) in DN [5]. Also, the aberrant expression of regulatory molecules such as the microRNA (miRNA), and lncRNA has been identified as a major determinant in the pathogenesis and progression of DN [6–11]. And the identification of these molecules and a comprehensive understanding of their supervisory mechanism and pathway in DN is very key to the development of a novel approach to the early detection, prognosis, and treatment of DN in patients.

Generally, the lncRNAs (with length > 200nt) constitute a major part of the ncRNAs and are deemed to be involved in the regulation of gene expression. For example, the ZFAS1 was reported to promote the tumorigenesis and development of colorectal cancer by regulating the DDX21-POLR1B axis, while its aberrant expression is associated with prognosis and chemosensitivity in cervical cancer [12, 13]. However, the functional effect and underlying mechanism of lncRNA on DN progression have yet to be further investigated.

Thus, the purpose of this study was to determine the involvement of ZFAS1 in the development and progression of DN and its probably regulatory mechanism with the hope that the result could help to further understand the pathogenesis of the disease and also the development of effective treatment for it. Using Lncbase, we predicted that ZFAS1 targets miR-588 in DN which abnormal expression has been reported to be involved in the pathogenesis of prostate cancer [14], human breast cancer [15], colorectal cancer [16], hepatocellular carcinoma [17], and osteosarcoma [18], but not in DN. Similarly, Targetscan predicted that miR-588 targets ROCK1 mRNA. We confirmed that ZFAS1 regulates HGMC proliferation, oxidative stress, fibrosis, and inflammation in HG-induced DN through the miR-588/ROCK1 axis and could be a potential diagnostic and prognostic tool, and also a therapeutic target for the prevention and treatment of DN.

## Materials and methods

### Serum samples collection

Blood samples that were gotten from DN (n = 20) and normal healthy (n = 20) individuals at the Affiliated Hospital of Qingdao University were spun at 2000×*g* for 10 min to obtain the serum, and were kept in liquid nitrogen at − 80 °C for further analysis. The study was conformed to the standard by the Declaration of Helsinki. This study was approved by the Medical Ethics Committee of the Affiliated Hospital of Qingdao University (Approval No. KE-2019–2201). Informed consent was obtained from each patient before enrollment in this study.

### Cell culture and induction of high glucose

Human glomerular mesangial cells (HGMC) were bought from Sciencell Research Laboratories (Carlsbad, CA, USA). Dulbecco’s modified Eagle’s medium (DMEM, Invitrogen, USA) comprising 10% FBS (Gibco, USA) was used to cultured HGMC cells (Ximbio, USA) with 5% CO_2_ at 37 °C. HGMC cells were cultured under normal glucose condition (Normal, 5.5 mM glucose) or HG condition (30 mM glucose) for 96 h. HG treatment was performed to mimic the DN cells.

### Cell transfection

MiR-588 inhibitors and the negative controls (miR-NC) were acquired from RiboBio (China). SiRNA targeting lncRNA ZFAS1 sequence (si-ZFAS1) was used for silencing the expression of lncRNA ZFAS1 in this study (GeneChem, China). The transfection was done using Lipofectamine 2000 reagent according to the manufacturer’s instructions (Invitrogen, USA). After, the cellular effect of this transfection was investigated through various molecular experiments such as cell viability and inflammatory cytokine production. For ROCK1 overexpression, the CDS sequencing of ROCK1 was constructed into pcDNA-3.1 (GeneChem, China) and transfected into HGMCs cells by using Lipofectamine 2000.

### Intracellular reactive oxygen species (ROS), superoxide dismutase (SOD), and malondialdehyde (MDA) Estimation

HGMCs ROS contents were evaluated using DCF fluorescence (Sigma-Aldrich) at a wavelength of 525 nm. The activity of SOD and level of MDA was estimated in the cellular supernatant with a commercial kit (Nanjing Institute of Bioengineering, China) at the wavelength of 412 nm in accordance with the manufacturer’s instruction.

### Quantitative real‑time polymerase chain reaction (qRT‑PCR)

HGMC RNA was extracted with TRIzol (Invitrogen, USA). After, reverse transcription was used to synthesize cDNA using HiScript QRT Super Mix (Vazyme, USA). The qRT-PCR analysis was done with a 7500 Real-Time PCR System (Applied Biosystems. USA) using the SYBR premix Ex TaqIIkit (TaKaRa, China) measured by using the 2^−ΔΔCt^ method. GAPDH was the endogenous control for ZFAS1, ROCK1, and U6 was the endogenous control of miR-588. The primers utilized in this experiment were gotten from GenePharma (Shanghai, China).

### Cell viability analysis

The cell viability assay was performed with CCK-8 in accordance with the manufacturer’s instructions (Dojindo, Japan). Briefly, approximately 5 × 10^3^ cells were seeded in 96-well plates, 10 µL CCK-8 solution (Solarbio, Beijing China) was added and incubated for 2 h at 37 °C. subsequently, the Microplate Reader (Bio-Rad) was employed to estimate the optical density at 450 nm.

### Enzyme-linked immunosorbent assay (ELISA)

After 48 h of culture, the cell culture supernatants were obtained to evaluate the levels of inflammation-related factors TNF-α, IL-1β, and -6 using the ELISA kit (R&D Systems, USA) and the Microplate Reader (Bio-Rad) at 450 nm.

### Western blot assay

Protein extraction was done with RIPA buffer (Beyotime. China). The concentration was evaluated, denatured, and separated by SDS-PAGE. It was then transferred to PVDF membranes (Beyotime) and blocked with milk (5%) for 2 h before incubation overnight at 4 °C with primary antibodies (1:1000) against Fibronectin (FN), Collagen type IV, and ROCK1 and GAPDH. After, the membranes were incubated with HRP-conjugated secondary antibody (1:1000) for 1 h. The proteins were visualized with BeyoECL Moon (Beyotime, China).

### Luciferase reporter assay

Luciferase reporter assay was performed in HGMC cells. First, ZFAS1 and ROCK1 fragments with (Wild type-WT) or without (Mutant type-MUT) the predicted miR-588 binding sites were introduced into pGl3 plasmids (Promega, USA). Next, for 48 h, co-transfection of the recombinant vectors, miR-588, and miR-NC into HGMC cells using Lipofectamine 2000 (Invitrogen. USA) was performed, and Dual-Luciferase Reporter Assay System (Promega) was employed to analyze the resulting Luciferase activity.

### RNA Immunoprecipitation (RIP) and RNA pull‑down assay

Detection of RIP was finished with Magna RIP RNA Binding Protein Immunoprecipitation Kit (Millipore, USA). ZFAS1 and miR-588 nucleotide sequence were used to treat HMGC cells which were later lysed with RIP lysis buffer comprising the protease inhibitors. Subsequently, overnight, the addition of IgG and Ago2 antibody (Abcam) to the cell lysates was performed at 4 °C. Then, immunoprecipitated RNAs were acquired. The evaluation of levels of ZFAS1 and miR-588 was done with qRT-PCR analysis. Transfection of HGMC cells with biotin-labeled Bio-miR-NC and Bio-miR-588 (WT and MUT) was also performed. After 48 h of transfection, cells were removed, and the collection of bound RNA was done with Pierce™ Magnetic RNA Pull-Down Kit (Thermo Fisher Scientific) in accordance with the guide. Lastly, ZFAS1 enrichment in the biotin-coupled miRNAs needed to be measured with qRT-PCR.

### Statistical analysis

All experiments were performed in triplicate. Data were analyzed with GraphPad Prism 6.0 (CA, USA) and expressed as the mean ± standard deviation. For comparisons, the Student’s *t*-test was employed and ANOVA together with Turkey’s test. The Spearman rank correlation coefficient was performed to identify the correlation between miR-588 and ZFAS1 expression. *p*-values < 0.05 were taken to be significant statistically.

## Results

### ZFAS1 is highly expressed in DN blood samples and HG-induced HGMC

**Fig. 1:**
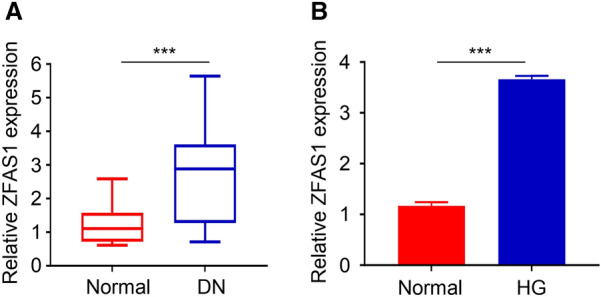
The expression of ZFAS1 in DN blood samples and HG-induced HGMC (**A** and **B**) ZFAS1 is highly upregulated DN samples and HG-induced HGMC compared to the normal healthy or control group

At the initial stage of this study, blood samples were collected from 20 diabetics nephropathy patients and 20 normal healthy people. After, qRT-PCR was used to determine the ZFAS1 differential expression between the two groups. The result revealed that ZFAS1 is highly upregulated in DN patients compared to the normal people’s sample and might be involved in the genesis of DN (*p* < 0.001, Fig. 1A). Furthermore, a DN cell model was built by inducing HGMC with high (25 mM) glucose concentration while the normal control cell group was induced with 5.5 mM of glucose. QRT-PCR analysis of ZFAS1 expression in the glucose-induced cells showed that ZFAS1 is significantly expressed in the high-glucose-induced (HG) cell than the normal group (*p* < 0.001, Fig. 1B).

### ZFAS1 regulates HG-induced proliferation, oxidative stress, fibrosis, and inflammation in mesangial cells

**Fig. 2:**
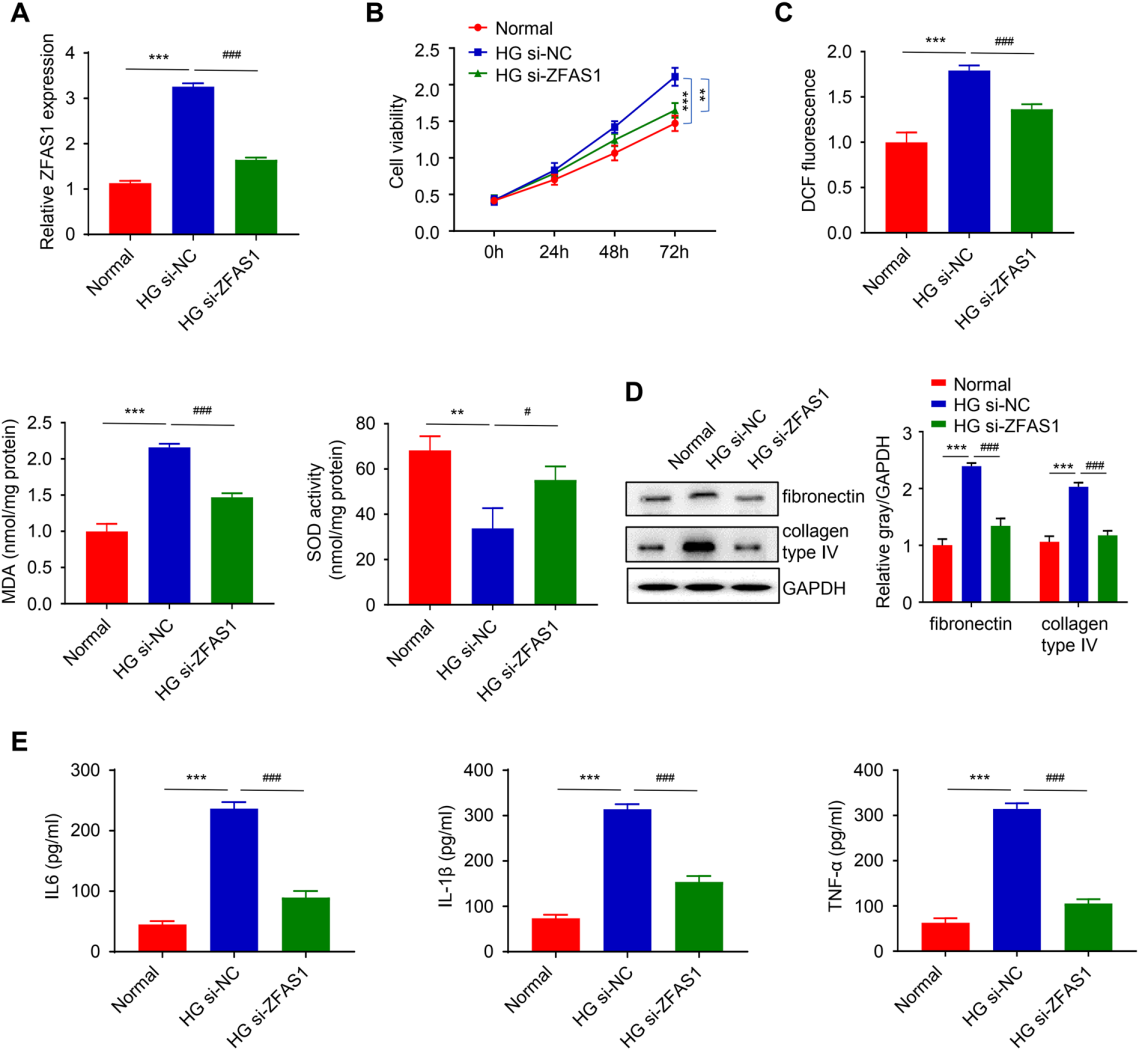
The lncRNA ZFAS1 regulates HG-induced proliferation, oxidative stress, fibrosis, and inflammation in mesangial cells **A** ZFAS1 expression was significantly inhibited in the HG-induced cell, after silencing ZFAS1. Silencing ZFAS1 significantly inhibited the proliferation of the HG-induced cell compared to the negative control group (**B**). Oxidative stress induced after ZFAS1 knockdown in HG-treated HGMC. The level of ROS and MDA were significantly reduced in the HG-treated cell relative to the HG-si-NC group while the SOD activity significantly increased (**C**). The western blot analysis result showed that the protein expression level of fibronectin and collagen type IV was significantly inhibited in the HG si-ZFAS relative to the control HG si-NC group (**D**). The level of inflammatory cytokines IL-6, IL-1β, and TNF-α, produced by HG-induced cells after ZFAS1 knockdown. The inflammatory cytokines were significantly downregulated after ZFAS1 knockdown (**E**). All the experimental data are shown as mean ± SD of three independent experiments and the significance level was defined as *p* < 0.05 (**p < 0.01 and *** *p* < 0.001 vs. normal group; ^#^*p* < 0.05, ^##^*p* < 0.01, and ^###^*p* < 0.001 vs. HG-si-NC group)

Further, glucose-induced cells were transfected with si-NC and si-ZFAS1 to examine the biological function of ZFAS1 in the DN. As showed in Fig. 2A the relative expression of ZFAS1 was significantly inhibited in the HG-induced cell, after knockdown with si-ZFAS1, compared to the negative control group (HG si-NC) (*p* < 0.001). Moreover, silencing ZFAS1 significantly reduced the viability of the HG-treated cell in a time-dependent manner opposed to the negative control group (HG si-NC) (*p *< 0.01, Fig. 2B). We reviewed the level of oxidative stress induced in the HG-treated cell, after silencing ZFAS1, by measuring the expression of respective oxidative stress-related markers including ROS, MDA, and SOD. Our results revealed that the level of ROS (as measured by DCF fluorescence) and MDA in the HG-treated cell were significantly reduced relative to the HG-si-NC (control) group, after silencing ZFAS1, while the SOD activity significantly increased (*p* < 0.001, Fig. 2C). Furthermore, western blot analysis was performed to detect the expression of the fibrogenesis-associated protein in HG-induced HGMC cells, after ZFAS1 knockdown. We found that the protein expression level of fibronectin and collagen type IV was significantly inhibited in the HG si-ZFAS relative to the control HG si-NC group (*p *< 0.001, Fig. 2D). Additionally, we measured the level of inflammatory cytokines IL-6, IL-1β, and TNF-α produced by the cells after HG induction and ZFAS knockdown through ELISA. The result shows that the induction of HGMC with HG consequently upregulated the level of inflammatory cytokines produced by the cell when compared to the normal HGMC group while silencing ZFAS1 significantly inhibited this (*p* < 0.001, Fig. 2E).

### ZFAS1 endogenously targets the miR-588 sequence

**Fig. 3:**
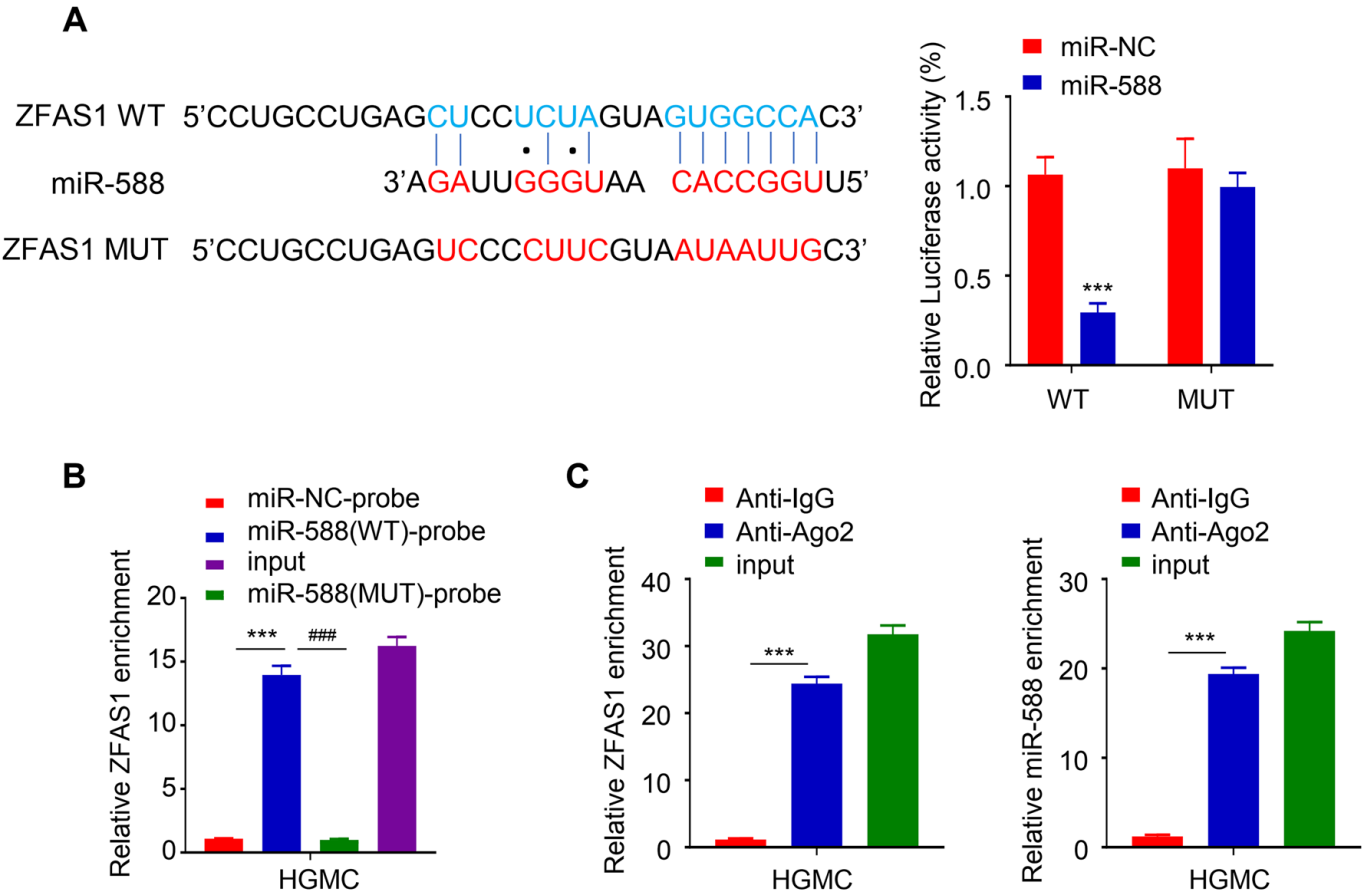
ZFAS1 endogenously targets the miR-588 sequence. Bioinformatics prediction of target miRNA. Lncbase predicted that the ZFAS1 targets miR-588 seed region sequences. Dual-luciferase reporter assay experiment. MiR-588 mimics significantly reduced the luciferase activity of HGMC co-transfected with the ZFAS1 wild type (WT) reporter plasmid but had no inhibitory effect on that of ZFAS1 mutant type (Mut) plasmid (**A**). Biotinylated RNA pull-down assay. Biotin-coupled probes with wild type (WT) miR-588 sequence pull-down more ZFAS1 than those with the mutated miR-588 sequence (MUT) (**B**). RIP-qRT-PCR analysis. More ZFAS1 and miR-588 were significantly enriched in the immunoprecipitated Ago2-containing complexes compared to the IgG group (**C**). Experiments were carried out in triplicates and *p* < 0.05 was chosen as the significance level (*** *p* < 0.001 and ^###^*p* < 0.001)

Using the lncbase database, we predicted that the ZFAS1 targets miR-588 seed region sequences (Fig. 3A). This was further confirmed through a dual-luciferase reporter assay which showed that miR-588 mimics significantly reduced the luciferase activity of HGMC that were co-transfected with the ZFAS1 wild type (WT) reporter plasmid compared to the control group (miR-NC) but had no inhibitory effect on the luciferase activity of the cell after mutating the predicted binding site of miR-588 in the ZFAS1 mutant type (Mut) plasmid vector (*p *< 0.001, Fig. 3A). Furthermore, through biocatalyst RNA pull-down assay, we confirmed that biotin-coupled probes with wild type (WT) miR-588 sequence could pull-down more ZFAS1 than the one with the mutated miR-588 sequence (MUT) (*p* < 0.001, Fig. 3B). We also found that more ZFAS1 and miR-588 were significantly enriched in immunoprecipitated Ago2-containing complexes compared to the IgG group, validating the ZFAS1-miR-588 ability to bind through the RISC complex (*p* < 0.001, Fig. 3C).

### MiR-588 regulates HG-induced proliferation, oxidative stress, fibrosis, and inflammation in mesangial cells

**Fig. 4:**
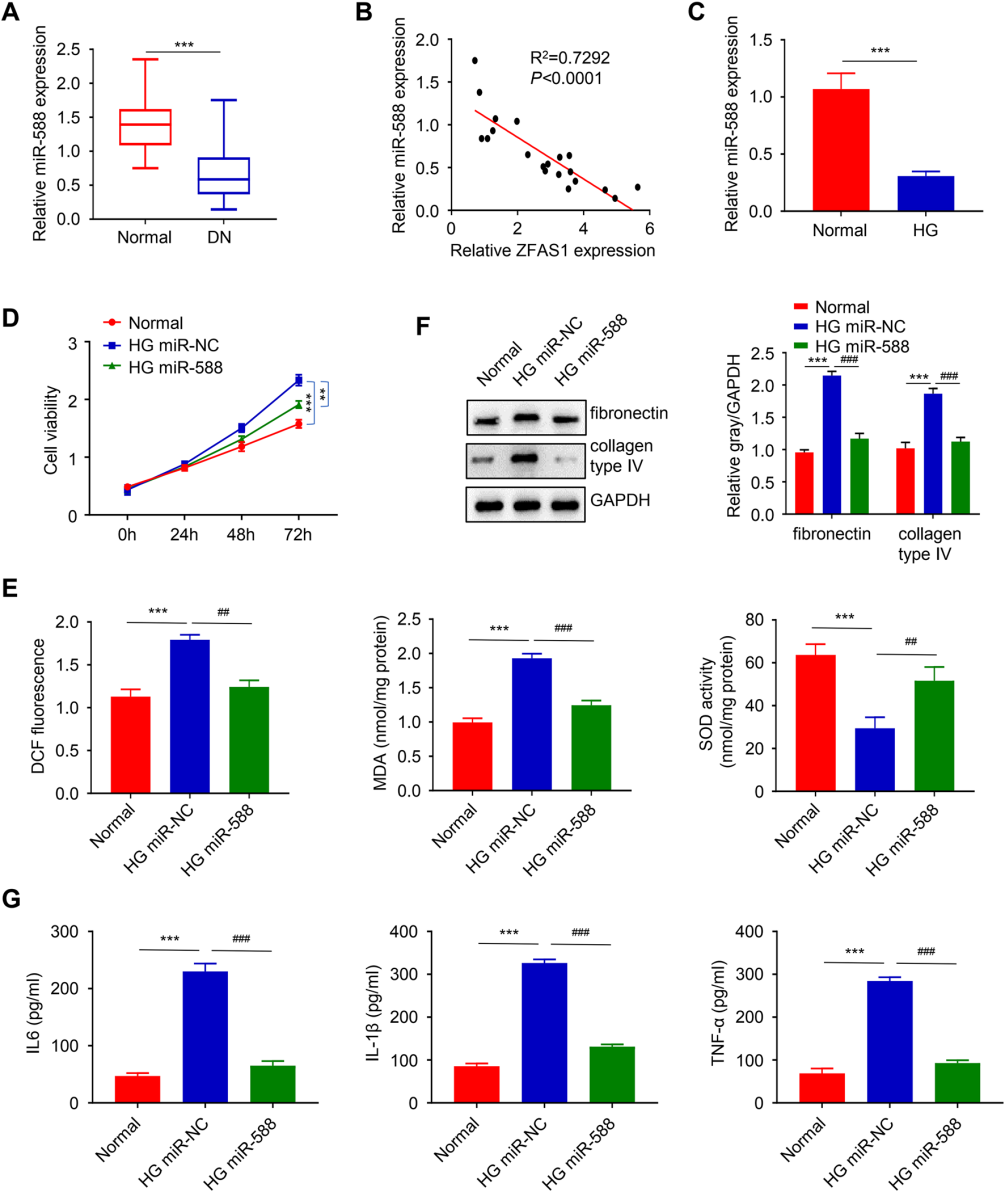
MiR-588 regulates HG-induced proliferation, oxidative stress, fibrosis, and inflammation in mesangial cells. The expression level of miR-588 in DN and HG-induced HGMC was significantly downregulated compared to the healthy or control group and was inversely related to ZFAS1 expression (**A**–**C**). CCK-assay and western blot analysis revealed the overexpression of miR-588 in HG-induced HGMC significantly reduced the cell viability, and the fibronectin and collagen type IV protein expression level, compared to the negative control group (**D** and **E**). overexpression of miR-588 markedly reversed the HG-induced upregulation of ROS and MDA, and downregulation of SOD, in the HG miR-NC treatment group (**F**). The overexpression of miR-588 significantly reduced the level of the HG-induced inflammatory cytokines secreted (**G**). Significance level was defined as *p*< 0.05 (**p < 0.01 and *** *p*< 0.001 vs. normal group; ^##^*p*< 0.01, and ^###^*p*< 0.001 vs. HG-si-NC group)

The expression level of miR-588 in DN blood samples was subsequently measured in this study through qRT-PCR. The result shows that miR-588 is significantly downregulated in DN samples relative to the normal ones and was inversely related to ZFAS1 expression (*p *< 0.001, Fig. 4A; and *p *< 0.0001, Fig. 4B). The further consequence shows that miR-588 expression is significantly repressed in HGMC after HG-induction (*p*< 0.001, Fig. 4C). On the other hand, we found that the overexpression of miR-588 in HG-induced HGMC significantly reduced the cell viability compared to miR-NC and also the fibronectin and collagen type IV protein expression (*p* < 0.01, Fig. 4D; and *p*< 0.001, Fig. 4E). Moreover, miR-588 upregulation markedly reversed the HG-induced upregulation of ROS and MDA, and downregulation of SOD, in the HG miR-NC treatment group (*p*< 0.001, Fig. 4F). Similarly, the overexpression of miR-588 significantly reduced the level of inflammatory cytokines secreted after HG-induction (*p*< 0.001, Fig. 4G).

### ROCK1 mRNA was a direct target of miR-588

**Fig. 5:**
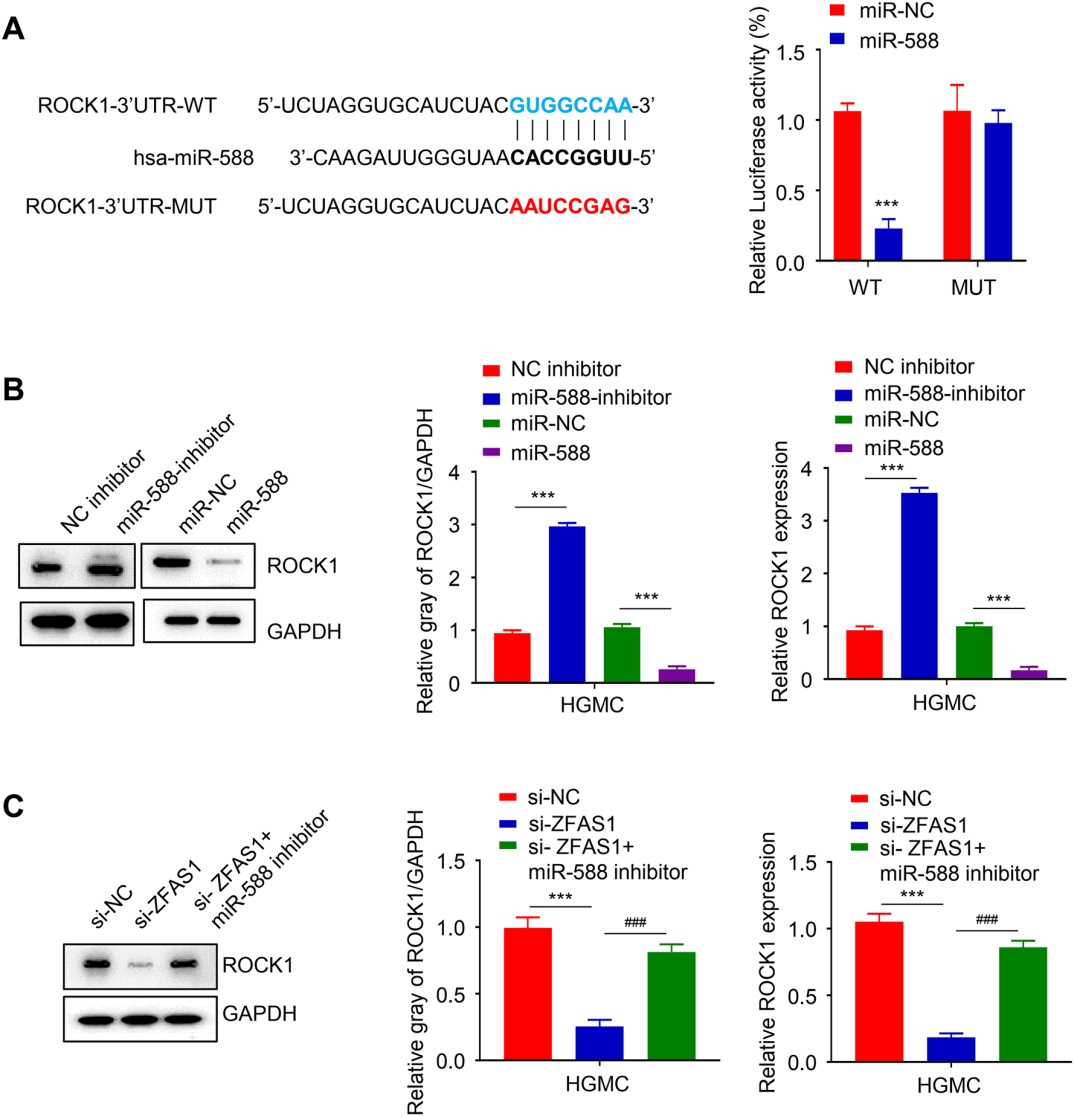
ROCK1 mRNA was a direct target of miR-588. Targetscan prediction of miR-588 target mRNA. Dual-luciferase reporter experiments. MiR-588 could significantly inhibit the luciferase activity of the HGMC co-transfected with the wild type (WT) ROCK reporter plasmid but had no inhibiting effect on that of the mutant type (MUT) ROCK group (**A**). QRT-PCR and Western blot analysis. Inhibiting miR-588 markedly upregulated the mRNA and protein expression level of ROCK in the HGMC compared to the negative control group while the overexpression of miR-588 reversed this (**B**). Inhibiting miR-588 restored si-ZFAS1-downregulated expression of ROCK1 mRNA and protein in the HGMC cell (**C**). (*** *p* < 0.001 ^###^*p* < 0.001)

We also predicted the presence of miR-588 binding sites in the 3’UTR region of ROCK using Target scan software and performed dual-luciferase reporter gene experiments in HGMC cells to confirm the prediction result (Fig. 5A). Results show that miR-588 could significantly inhibit the luciferase activity of the HGMC group that was co-transfected with the wild type (WT) ROCK reporter plasmid, compared to the miR-NC, while it had no notable inhibiting effect on the luciferase activity of the cells co-transfected with the mutant type (MUT) ROCK reporter plasmid (*p *< 0.001, Fig. 5A). Furthermore, through qRT-PCR and Western blotting analysis, we observed that inhibiting miR-588 expression in the HGMC markedly upregulated the mRNA and protein expression level of ROCK compared to the negative control group (NC inhibitor), while the overexpression of miR-588 significantly reduced the ROCK expression (*p *< 0.001, Fig. 5B). Similarly, inhibiting miR-588 in the HGMC cell restored si-ZFAS1-downregulated expression of ROCK1 mRNA and protein (*p*< 0.001, Fig. 5C).

### ZFAS1 inhibits HG-induced proliferation, oxidative stress, fibrosis, and inflammation in mesangial cells via the miR-588/ROCK1 axis

**Fig. 6:**
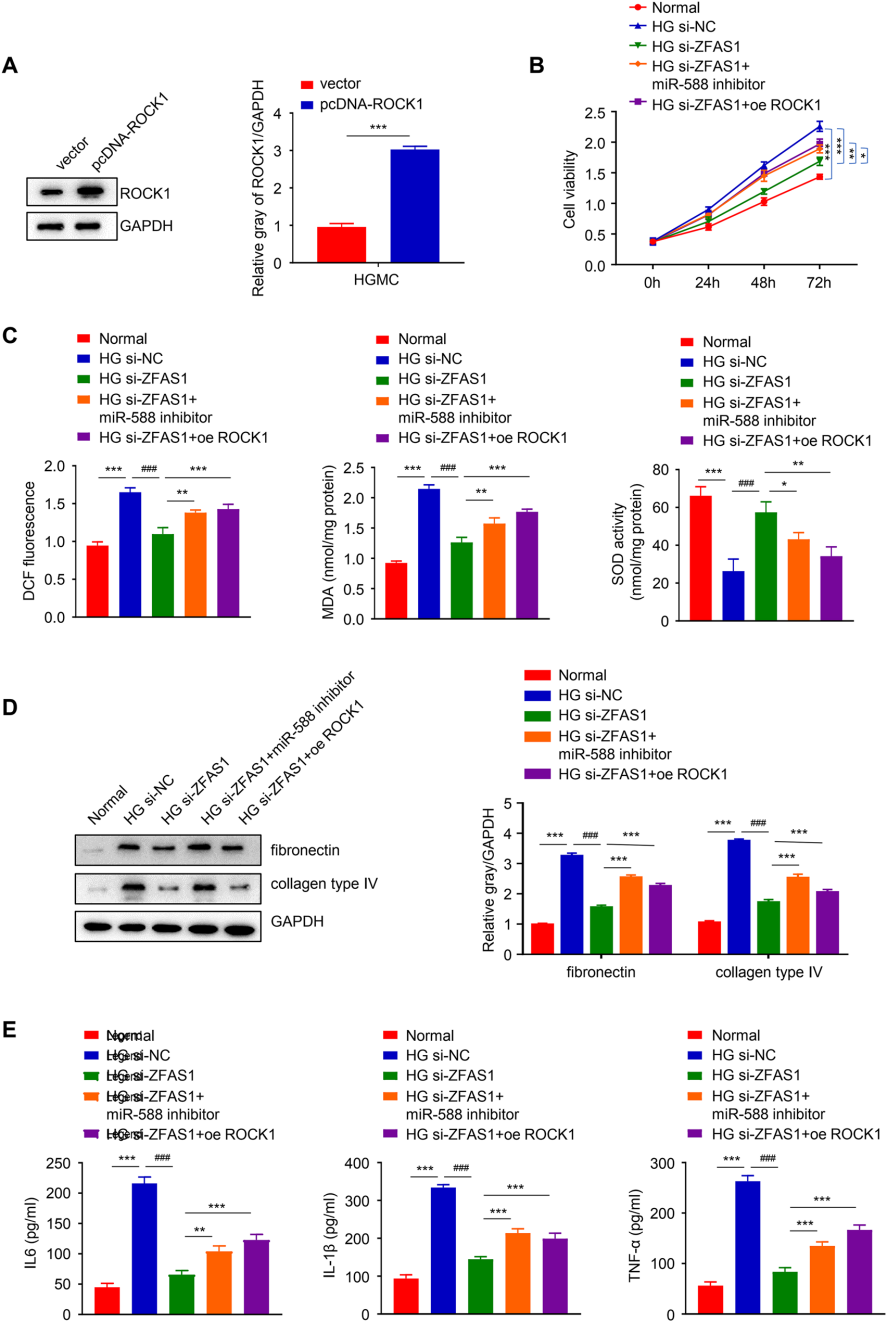
ZFAS1 inhibits HG-induced proliferation, oxidative stress, fibrosis, and inflammation in mesangial cells via the miR-588/ROCK1 axis. Transfecting pcDNA-ROCK1 overexpression plasmid into HGMC significantly upregulated ROCK1 expression (**A**). The overexpression of ROCK1 restored HG-induced cell viability that was reduced by the depleted ZFAS1 (**B**). MiR-588 inhibition or overexpression of ROCK1 partly restored the depleted level of ROS and MDA and repressed the SOD activity that was increased by ZFAS1 knockdown (**C**). MiR-588 inhibitor or overexpressed ROCK1 also recovered the downregulated fibronectin and collagen type IV protein expression that was induced by ZFAS1 knockdown in the HGMC (**D**). The depleted level of IL-6, IL-1β, and TNF-α after ZFAS1 knockdown in the HG-induced cell was also recovered by miR-588 inhibition or ROCK1 overexpression (**E**). Significance level was set at *p* < 0.05 (**p < 0.01, *** *p* < 0.001; ^###^*p* < 0.001)

To confirm if ZFAS1 can inhibit the proliferation, oxidative stress, fibrosis, and inflammation of mesangial cells through the miR-588/ROCK1 axis, the HG-induced HGMC was co-transfected with ROCK1 overexpression plasmid. As showed in Fig. 6A the ROCK1 protein expression was significantly upregulated in the HGMC after transfection with pcDNA-ROCK1, confirming the efficacy of the ROCK1 overexpression plasmid used for the experiment (*p *< 0.001). Our functional analysis experiment result showed that the overexpression of ROCK1 restored HG-induced cell viability that was diminished by the depleted ZFAS1 (*p *< 0.01, Fig. 6B). Consistently, the inhibition of miR-588 or overexpression of ROCK1 partly restored the depleted level of ROS and MDA and repressed the SOD activity that was increased by ZFAS1 knockdown (*p* < 0.001, Fig. 6C). Subsequent assessment of HG-induced cell fibrosis revealed that miR-588 inhibitor or overexpressed ROCK1 recovered the downregulated fibronectin and collagen type IV protein expression that was induced by silencing ZFAS1 in the cell, suggesting that the inhibition of miR-588 or the overexpression ROCK1 could increase cell fibrosis in DN (*p* < 0.001, Fig. 6D). Also, the depleted level of IL-6, IL-1β, and TNF-α after knocking down ZFAS1 in the HG-induced cell was also recovered by miR-588 inhibition or ROCK1 overexpression (**p < 0.01  ***p < 0.001, Fig. 6E). Altogether, these data suggest that ZFAS1 inhibits the proliferation, oxidative stress, fibrosis, and inflammation of HG-induced mesangial cells through the miR-588/ROCK1 axis and its knockdown might be an effective therapeutic approach to DN treatment.

## Discussion

DN is one of the complex diseases with diverse pathogenesis. Despite years of research, its development is not yet fully understood. Therefore, in this research, we provided, for the first time, evidence that the ZFAS1 regulates the proliferation, oxidative stress, fibrosis, and inflammation of HG-induced DN through the miR-588/ROCK1 axis. Numerous studies have shown that the ncRNAs are involved in the progression of DN and might be useful therapeutic targets for DN treatment [19]. For instance, the circRNA_010383 has been reportedly shown to promote proteinuria and cause the exacerbation of renal fibrosis in DN by sponging the miRNA-135a [10], while the lncRNA NR_038323 acts as a tumor suppressor in DN and suppresses renal fibrosis by endogenously targeting the miR-324-3p/DUSP1 axis [20]. Specifically, the overexpression of ZFAS1 has been shown to promote the proliferative, invasive, and metastatic ability of prostate cancer cells by regulating miR-135a-5p expression [21]. Exosomal ZFAS1 has also been declared to regulate the proliferation, invasion, migration, and apoptosis of esophageal squamous cell carcinoma by regulating the miRNA-124/STAT3 axis [22]. However, its role in DN has not been reported. In our study, ZFAS1 was found to be significantly upregulated in DN blood samples, and also in HG-induced cells, which suggest that the ZFAS1 could serve as an effective biomarker or potential therapeutic target for treating DN.

Furthermore, functional study result shows that ZFAS1 knockdown significantly inhibited the HG-induced HGMC proliferation and also aggravated its oxidative stress as indicated by the decline in the ROS and MDA level, and also the significant increase in SOD activity. Accumulating report shows that the excessive proliferation of HGMC is a critical characteristic of DN [23]. Besides, HG-induced oxidative stress injury in HGMC has been recognized as a major contributor to the pathogenesis and development of DN [24, 25]. In line with previous studies [26, 27], we found that the level of accumulated extracellular matrix (ECM) protein-fibronectin and collagen type IV, and inflammatory cytokines produced after ZFAS1 knockdown was markedly reduced, further suggesting a therapeutic potential of ZFAS1. To understand the ZFAS1 molecular mechanism of regulation in DN, target miRNA and mRNA were predicted and validated through a dual-luciferase reporter assay. The result showed that ZFAS1 endogenously sponges miR-588 through a regulatory ceRNA network, while miR-588 functions in the post-transcription inhibition of ROCK1 mRNA. The miR-588 acts as an anti-oncogene in lung squamous cell carcinoma by targeting the progranulin mRNA, inhibiting the migration and invasion of the cell [28]. Its downregulation has also been reported to promote gastric cancer cell progression [29]. On the other hand, ROCK1 has also been declared to play an important role in DN [30, 31]. Its inhibition has has been demonstrated mainly block endothelial-to-mesenchymal transition, reduce glomerular endothelial permeability, and albuminuria in DN [30]. The inhibition of the RhoA/ROCK1 pathway by 3-Hydroxy-3-methylglutaryl CoA reductase inhibitor has also been reported to block the HG-induced dysregulation of occludin and ZO-1 and could prevent the onset of albuminuria during the early stage of DN [32]. Moreover, our rescue experiment confirmed that miR-588 inhibition and the overexpression of ROCK1 could partially reverse the protective effect of ZFAS1 knockdown on the HG-induced proliferation, oxidative stress, fibrosis, and inflammation in HGMC. In summary, in this study, we focused on the ZFAS1 regulatory role and mechanism in the progression of DN and found that ZFAS1 regulates the proliferation, oxidative stress, fibrosis, and inflammation of HG-induced DN by downregulating miR-588 expression and upregulating ROCK1 mRNA and protein expression. ZFAS1 could be a potential diagnostic and prognostic target for DN and also a therapeutic target for its treatment.

## Conclusions

The lncRNA ZFAS1 regulates the proliferation, oxidative stress, fibrosis, and inflammation of high glucose-induced diabetic nephropathy through the miR-588/ROCK1 axis. The ZFAS1 might be a novel biomarker for the early diagnosis, treatment, and prognosis of diabetic nephropathy in patients.

## Data Availability

The data used or analyzed during this study are included in this article and available from the corresponding author upon reasonable request.

## References

[1] Zhang X-X, Kong J, Yun K (2020). Prevalence of diabetic nephropathy among patients with type 2 diabetes mellitus in China: a meta-analysis of observational studies. J Diabetes Res.

[2] Umanath K, Lewis JB (2018). Update on diabetic nephropathy: core curriculum 2018. Am J Kidney Dis.

[3] Ritz E, Rychlík I, Locatelli F, Halimi S (1999). End-stage renal failure in type 2 diabetes: a medical catastrophe of worldwide dimensions. Am J Kidney Dis.

[4] Xu B-H, Sheng J, You Y-K, Huang X-R, Ma RCW, Wang Q (2020). Deletion of Smad3 prevents renal fibrosis and inflammation in type 2 diabetic nephropathy. Metab Clin Exp.

[5] Jiang N, Zhao H, Han Y, Li L, Xiong S, Zeng L (2020). HIF-1α ameliorates tubular injury in diabetic nephropathy via HO-1-mediated control of mitochondrial dynamics. Cell Prolif.

[6] Long J, Badal SS, Ye Z, Wang Y, Ayanga BA, Galvan DL (2016). Long noncoding RNA Tug1 regulates mitochondrial bioenergetics in diabetic nephropathy. J Clin Invest.

[7] Loganathan TS, Sulaiman SA, Murad NAA, Shah SA, Abdul Gafor AH, Jamal R (2020). Interactions among non-coding RNAs in diabetic nephropathy. Front Pharmacol..

[8] Hu M, Wang R, Li X, Fan M, Lin J, Zhen J (2017). LncRNA MALAT1 is dysregulated in diabetic nephropathy and involved in high glucose-induced podocyte injury via its interplay with β-catenin. J Cell Mol Med.

[9] Delić D, Eisele C, Schmid R, Baum P, Wiech F, Gerl M (2016). Urinary exosomal miRNA signature in type II diabetic nephropathy patients. PLOS ONE.

[10] Peng F, Gong W, Li S, Yin B, Zhao C, Liu W (2020). CircRNA_010383 acts as a sponge for miR-135a and its downregulated expression contributes to renal fibrosis in diabetic nephropathy. Diabetes..

[11] Hu W, Han Q, Zhao L, Wang L (2019). Circular RNA circRNA_15698 aggravates the extracellular matrix of diabetic nephropathy mesangial cells via miR-185/TGF-β1. J Cell Physiol..

[12] Wang X, Wu Z, Qin W, Sun T, Lu S, Li Y (2020). Long non-coding RNA ZFAS1 promotes colorectal cancer tumorigenesis and development through DDX21-POLR1B regulatory axis. Aging.

[13] Feng L-L, Shen F-R, Zhou J-H, Chen Y-G (2019). Expression of the lncRNA ZFAS1 in cervical cancer and its correlation with prognosis and chemosensitivity. Gene.

[14] Retraction. microRNA-588 is upregulated in human prostate cancer with prognostic and functional implications. J Cell Biochem. 2019;120:12070. 10.1002/jcb.26417. 10.1002/jcb.26417 28980707

[15] Yu M, Zhang X, Li H, Zhang P, Dong W (2017). MicroRNA-588 is downregulated and may have prognostic and functional roles in human breast cancer. Med Sci Monit.

[16] Fattahi F, Kiani J, Khosravi M, Vafaei S, Mohammadi A, Madjd Z (2019). Enrichment of up-regulated and down-regulated gene clusters using gene ontology, miRNAs and lncRNAs in colorectal cancer. Comb Chem High Throughput Screening.

[17] Liu Z, Mo H, Sun L, Wang L, Chen T, Yao B (2020). Long noncoding RNA PICSAR/miR-588/EIF6 axis regulates tumorigenesis of hepatocellular carcinoma by activating PI3K/AKT/mTOR signaling pathway. Cancer Sci.

[18] Liu R, Ju C, Zhang F, Tang X, Yan J, Sun J (2020). LncRNA GSEC promotes the proliferation, migration and invasion by sponging miR-588/ EIF5A2 axis in osteosarcoma. Biochem Biophys Res Commun.

[19] Yan B, Tao Z-F, Li X-M, Zhang H, Yao J, Jiang Q (2014). Aberrant expression of long noncoding RNAs in early diabetic retinopathy. Invest Opthalmol Vis Sci.

[20] Ge Y, Wang J, Wu D, Zhou Y, Qiu S, Chen J (2019). lncRNA NR_038323 suppresses renal fibrosis in diabetic nephropathy by targeting the miR-324-3p/DUSP1 axis. Mol Ther Nucleic Acids.

[21] Pan J, Xu X, Wang G (2020). lncRNA ZFAS1 is involved in the proliferation, invasion and metastasis of prostate cancer cells through competitively binding to miR-135a-5p. Cancer Manag Res.

[22] Li Z, Qin X, Bian W, Li Y, Shan B, Yao Z (2019). Exosomal lncRNA ZFAS1 regulates esophageal squamous cell carcinoma cell proliferation, invasion, migration and apoptosis via microRNA-124/STAT3 axis. J Exp Clin Cancer Res.

[23] Chen C, Lin J, Li L, Zhu T, Gao L, Wu W (2019). The role of the BMP4/Smad1 signaling pathway in mesangial cell proliferation: a possible mechanism of diabetic nephropathy. Life Sci.

[24] Wang Y, He Z, Yang Q, Zhou G (2019). XBP1 inhibits mesangial cell apoptosis in response to oxidative stress via the PTEN/AKT pathway in diabetic nephropathy. FEBS Open Bio.

[25] Mathur A, Pandey VK, Kakkar P (2018). Activation of GSK3β/β-TrCP axis via PHLPP1 exacerbates Nrf2 degradation leading to impairment in cell survival pathway during diabetic nephropathy. Free Radic Biol Med.

[26] Mason RM (2003). Extracellular matrix metabolism in diabetic nephropathy. J Am Soc Nephrol.

[27] Yang S, Zhang J, Wang S, Shi J, Zhao X (2017). Knockdown of angiopoietin-like protein 2 ameliorates diabetic nephropathy by inhibiting TLR4. Cell Physiol Biochem.

[28] Qian L, Lin L, Du Y, Hao X, Zhao Y, Liu X (2016). MicroRNA-588 suppresses tumor cell migration and invasion by targeting GRN in lung squamous cell carcinoma. Mol Med Rep.

[29] Chen Y, Zhang J, Gong W, Dai W, Xu X, Xu S (2020). miR-588 is a prognostic marker in gastric cancer. Aging.

[30] Peng H, Li Y, Wang C, Zhang J, Chen Y, Chen W (2016). ROCK1 induces endothelial-to-mesenchymal transition in glomeruli to aggravate albuminuria in diabetic nephropathy. Sci Rep.

[31] Rao J, Ye Z, Tang H, Wang C, Peng H, Lai W (2017). The RhoA/ROCK pathway ameliorates adhesion and inflammatory infiltration induced by AGEs in glomerular endothelial cells. Sci Rep.

[32] Peng H, Luo P, Li Y, Wang C, Liu X, Ye Z (2013). Simvastatin alleviates hyperpermeability of glomerular endothelial cells in early-stage diabetic nephropathy by inhibition of RhoA/ROCK1. PloS One.

